# Evolutionary history of the alpha2,8-sialyltransferase (ST8Sia) gene family: Tandem duplications in early deuterostomes explain most of the diversity found in the vertebrate ST8Sia genes

**DOI:** 10.1186/1471-2148-8-258

**Published:** 2008-09-23

**Authors:** Anne Harduin-Lepers, Daniel Petit, Rosella Mollicone, Philippe Delannoy, Jean-Michel Petit, Rafael Oriol

**Affiliations:** 1 Laboratoire de Glycobiologie Structurale et Fonctionnelle, CNRS, UMR 8576, Université des Sciences et Technologies de Lille, 59655 Villeneuve d'Ascq, France; 2 Laboratoire de Génétique Moléculaire Animale, INRA UMR 1061, Université de Limoges Faculté des Sciences et Techniques, 123 avenue Albert Thomas, 87060, Limoges, France; 3 Unité de Microenvironnement et physiologie de la différenciation, INSERM U602, Université de Paris Sud XI, 16 Avenue Paul Vaillant-Couturier, 94807, Villejuif, France

## Abstract

**Background:**

The animal sialyltransferases, which catalyze the transfer of sialic acid to the glycan moiety of glycoconjugates, are subdivided into four families: ST3Gal, ST6Gal, ST6GalNAc and ST8Sia, based on acceptor sugar specificity and glycosidic linkage formed. Despite low overall sequence identity between each sialyltransferase family, all sialyltransferases share four conserved peptide motifs (L, S, III and VS) that serve as hallmarks for the identification of the sialyltransferases. Currently, twenty subfamilies have been described in mammals and birds. Examples of the four sialyltransferase families have also been found in invertebrates. Focusing on the ST8Sia family, we investigated the origin of the three groups of α2,8-sialyltransferases demonstrated in vertebrates to carry out poly-, oligo- and mono-α2,8-sialylation.

**Results:**

We identified in the genome of invertebrate deuterostomes, orthologs to the common ancestor for each of the three vertebrate ST8Sia groups and a set of novel genes named ST8Sia EX, not found in vertebrates. All these ST8Sia sequences share a new conserved family-motif, named "C-term" that is involved in protein folding, via an intramolecular disulfide bridge. Interestingly, sequences from *Branchiostoma floridae *orthologous to the common ancestor of polysialyltransferases possess a polysialyltransferase domain (PSTD) and those orthologous to the common ancestor of oligosialyltransferases possess a new ST8Sia III-specific motif similar to the PSTD. In osteichthyans, we have identified two new subfamilies. In addition, we describe the expression profile of ST8Sia genes in *Danio rerio*.

**Conclusion:**

Polysialylation appeared early in the deuterostome lineage. The recent release of several deuterostome genome databases and paralogons combined with synteny analysis allowed us to obtain insight into events at the gene level that led to the diversification of the ST8Sia genes, with their corresponding enzymatic activities, in both invertebrates and vertebrates. The initial expansion and subsequent divergence of the ST8Sia genes resulted as a consequence of a series of ancient duplications and translocations in the invertebrate genome long before the emergence of vertebrates. A second subset of ST8sia genes in the vertebrate genome arose from whole genome duplication (WGD) R1 and R2. Subsequent selective ST8Sia gene loss is responsible for the characteristic ST8Sia gene expression pattern observed today in individual species.

## Background

Sialic acids (Neu5Ac, Neu5Gc, KDN) are negatively charged monosaccharides usually found at the non-reducing end of carbohydrate groups of animal glycoconjugates. Sialic acids occur widely in the deuterostome lineage (vertebrates, cephalochordates, ascidians, echinoderms) and they occasionally are encountered in protostomes (mollusks and arthropods) [1]. In vertebrates, sialic acids are either α2,3- or α2,6-linked to β-D-galactopyrannose (Gal), α2,6-linked to β-D-*N*-acetylglucosamine (GlcNAc) or β-D-*N*-acetylgalactosamine (GalNAc) or, α2,8-linked to another sialic acid forming mono-, oligo- or poly-α2,8-sialylated (PSA) chains (according to the degree of polymerization on glycoconjugates). The α2,8-linked polyNeu5Ac chain was first described in the polysialoglycoproteins (PSGPs) found in the cortical alveoli of unfertilized eggs of rainbow trout [2]. In mammals, PSA chains are primarily linked to the *N*-glycans of the neuronal cell adhesion molecule (N-CAM) and control the early developmental stages of the vertebrate embryo and neurogenesis (for a review see [3]). More recently, Guérardel *et al*. [4] described a unique oligo- and poly-sialylation pattern on glycoconjugates of zebrafish embryos suggesting that fine tuning of the PSA chain length is crucial for fertilization and development. In addition, several structural studies of glycoconjugates in a subset of sea urchin species demonstrated the presence of α2,8-polysialic acid chains. These observations raised the question of how far back in evolution can the α2,8-sialyltransferases be traced?

Despite low overall sequence identity, all the animal sialyltransferases catalyzing the biosynthesis of sialoglycoconjugates belong to CAZy glycosyltransferase-family 29 [5,6] and share four conserved peptide motifs called sialylmotifs L (large), S (small), III and VS (very small) [7-9]. These motifs are important for maintenance of the 3-D structure, substrate binding and catalysis [7,10-12]. Moreover, recent studies have identified linkage-specific sequence motifs (family motifs) in each of the four known sialyltransferase families (ST3Gal, ST6Gal, ST6GalNAc and ST8Sia), that are probably involved in determining linkage specificity and acceptor monosaccharide recognition [13]. Previously, we reported specific conserved amino acid positions that defined each of the twenty known vertebrate sialyltransferase subfamilies [14]. The enzymes of the ST8Sia family, which mediate the transfer of Neu5Ac to other Neu5Ac moieties found in glycoproteins and glycolipids are well described in some deuterostome lineages [15,16]. Partial redundancy of enzymatic activities among animal sialyltransferases suggests evolutionary flexibility allowing development of new animal lineages with new sialylated glycoconjugates with potentially new functions [17].

Since both uncharacterized sialyltransferases and new sialoglycoconjugates have been described in recent years, one of the major challenges facing glycobiologists is to determine the donor and acceptor specificities of each enzyme. Our phylogenetic analysis of the ST8Sia family suggests the existence of a set of divergent genes found only in the invertebrate deuterostomes *Strongylocentrotus purpuratus *and *Branchiostoma floridae *that we have named ST8Sia EX. We show that the majority of these ST8Sia EX genes arose as a result of tandem duplications, from an ancestral ST8Sia EX gene in the amphioxus lineage that was apparently lost in vertebrates. Among the remaining three groups of vertebrate ST8Sia genes, some subfamilies have emerged as a result of the whole genome duplications (WGD R1 and WGD R2) [18-25] and some subfamilies might have disappeared after massive gene loss [26,27]. Analysis of orthologous and paralogous relationships of these genes suggests that polysialylation initially appeared in the deuterostome lineage.

## Results

### Identification of ST8Sia sequences

**Figure 1:**
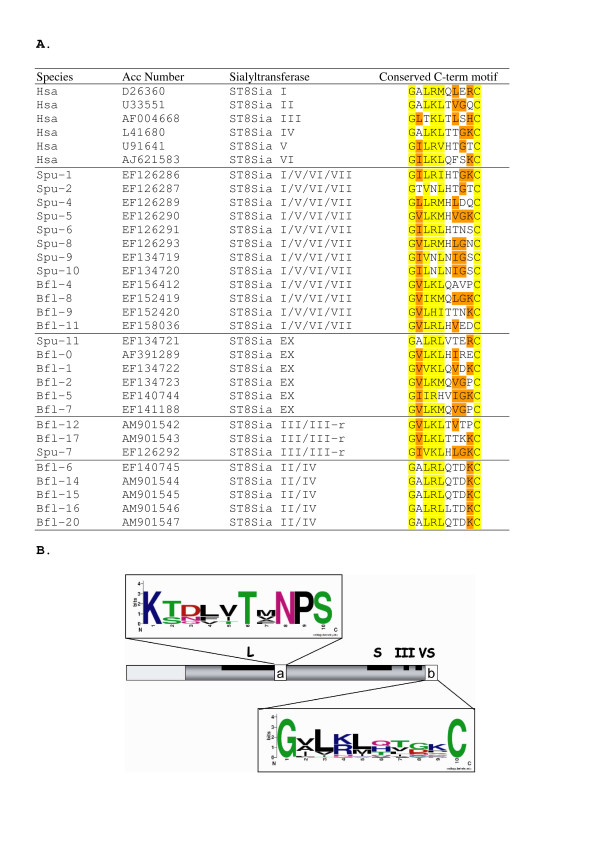
**A. Multiple sequence alignments of the vertebrate and invertebrate ST8Sia reveal a new family motif named C-term**. Black letters with a yellow background represent conserved positions at the 90% level for all the ST8Sia sequences whereas the orange background represents conserved positions at > 50%. **B. Sequence logo of the ST8Sia family motifs**. Relative positions in the ST8Sia sequences of the ST8Sia family-motif (a) described by Patel and Balaji [13] and the new C-term motif (b) found in all the ST8Sia sequences. In the logos, amino acids are colored according to their chemical properties: polar amino acids (G, C, S, T, Y) are green, basic (K, R, H) are blue, acidic (D, E) are red, hydrophobic (A, V, L, I, P, W, F, M) are black and neutral polar amino acids (N, Q) are pink. The overall height of the stack indicates the sequence conservation at a given position, while the height of symbols within the stack indicates the relative frequency of each amino at that position [69,70].

In order to identify putative genes encoding proteins with significant similarity to ST8Sia, we carried out BLAST search using the known vertebrate ST8Sia sequences. The search was based on the fact that the highly conserved sialylmotif peptide consensus sequences (L, S, III and VS) [14] are characteristic of all animal sialyltransferases and consequently serve as hallmarks for their identification. Thirty-five vertebrate ST8Sia sequences and twenty-seven invertebrate ST8Sia sequences from *Strongylocentrotus purpuratus *and *Branchiostoma floridae *were identified for the first time and are reported in additional file 1. However, due to very low sequence identity within the N-terminal region, the single transmembrane domain present in the vertebrate ST8Sia protein could not be identified in many of the invertebrate sequences. Nevertheless, we could delineate a new ST8Sia specific family-motif that we named "C-term" (Fig. 1). The cysteine residue of this motif forms a conserved intramolecular disulfide bridge with a second conserved cysteine residue located in sialylmotif L. This S-S bond is essential for correct folding and enzymatic activity of ST8Sia [28]. We assessed the orthology of vertebrate and invertebrate sequences by alignment with ClustalW, G-BLOCKS selection of informative positions and constructed maximum likelihood phylogenetic trees. We found that bony fishes such as zebrafish *Danio rerio*, medaka *Oryzias latipes*, 3-spined stickleback *Gasterosteus aculeatus*, tetraodonte *Tetraodon nigroviridis *and fugu *Takifugu rubripes *have orthologs of the other mammalian ST8Sia subfamilies (additional file 2). Moreover, two new subfamilies are present in a subset of bony fishes and are named ST8Sia III-related (ST8Sia III-r) because of their clear sequence relationship to the ST8Sia III subfamily and ST8Sia VII, respectively. Two ST8Sia VII genes are found in a head to tail arrangement on the same chromosome in the zebrafish genome suggesting tandem duplication of these genes. Consequently, we named these genes ST8Sia VIIA and ST8Sia VIIB.

**Figure 2:**
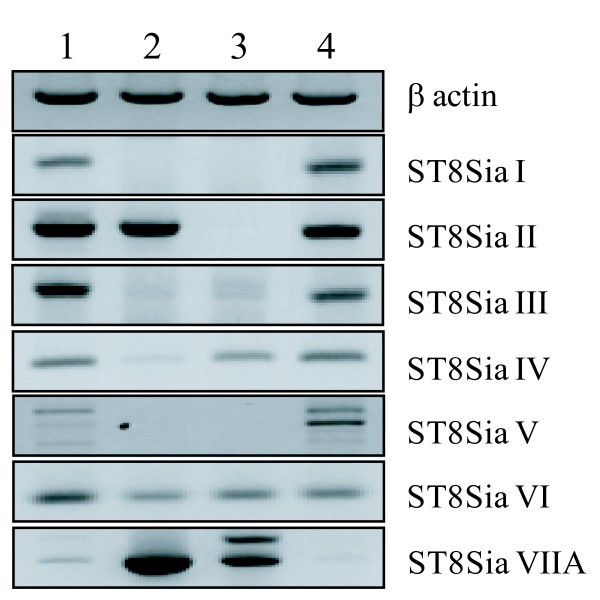
**Expression pattern of the zebrafish ST8Sia genes in various tissues, as determined by RT-PCR**. Lane 1, 36 h embryo; lane 2, ovary; lane 3, intestine; lane 4, brain. The zebrafish β-actin gene was used as a positive control of PCR amplification.

Because the greatest number of ST8Sia genes was found in zebrafish, we analyzed the expression patterns of the ST8Sia genes in some tissues of this species by RT-PCR (Fig. 2). All the ST8Sia genes were differentially transcribed in various *D. rerio *adult tissues and in the 36 h embryo. However, we did not detect a ST8Sia VIIB transcript, which is in agreement with the fact that ESTs corresponding to this gene were not found in the databanks.

### Phylogenetic analysis

**Figure 3:**
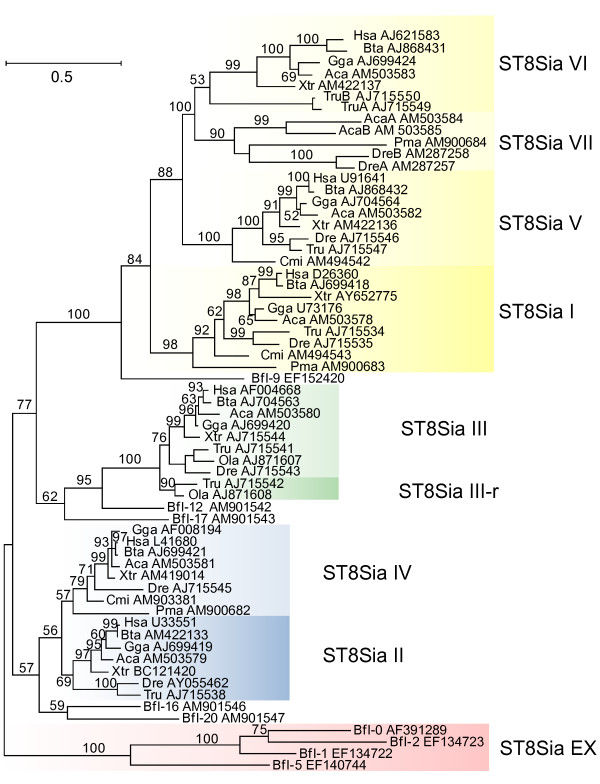
**Maximum Likelihood phylogenetic tree of 63 sialyltransferases of the ST8Sia family**. A Maximum Likelihood phylogenetic tree was constructed with the Phyml, JTT model of amino acid substitution; 63 ST8Sia sequences, 201 out of 426 positions (47%) were selected with G-BLOCKS. Bootstrap values were calculated from 500 replicates and values > 50% are reported at the left of each divergence point. GenBank accession numbers are indicated after the abbreviated name of the animal species. The tree was rooted with the invertebrate *B. floridae *sequences Bfl-0, Bfl-1, Bfl-2 and Bfl-5 as the outgroup.

Because of the high degree of similarity within each subfamily of the mammalian ST8Sia sequences, we used only the *Bos taurus *and *Homo sapiens *amino acid sequences for our initial phylogenetic analysis. Moreover, in order to conserve the greatest number of amino acid positions selected by G-BLOCKS [29] some *B. floridae *and all the *S. purpuratus *sequences were discarded due to their high sequence divergence. The maximum likelihood tree comprises four main branches supported by bootstrap values greater than 50% (Fig. 3).

The first branch, at the origin of the tree, grouped a series of sequences from the cephalochordate *B. floridae *(Bfl-0, Bfl-1, Bfl-2, Bfl-5) indicating that they might share a common ancestor. A second analysis including all the available ST8Sia sequences was performed with 173 G-BLOCKS selected positions. The resulting phylogenetic tree gave the same topology (additional file 3). Each of the main branches, except the group ST8Sia II/ST8Sia IV, possesses at least one ortholog in *S. purpuratus*.

To determine the most probable root of the ST8Sia family, we rooted the tree with the human sialyltransferase sequences belonging to the ST6GalNAc, ST6Gal and ST3Gal gene families (additional file 4). By multiple alignments, G-BLOCKS selection of informative positions, and maximum likelihood tree construction, the topology always confirmed the basal position of the ST8Sia EX group within the ST8Sia family. Consequently, it would appear that this ST8Sia EX group has evolved by multiple duplication events in the cephalochordate *B. floridae *and the echinoderm *S. purpuratus*, but has disappeared in vertebrates (Fig. 3 and additional file 3).

**Figure 4:**
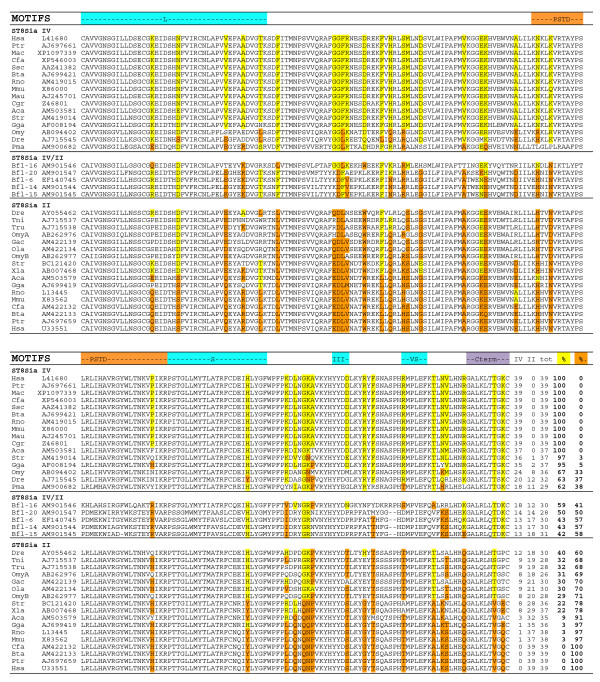
**ClustalRO alignment of all the ST8Sia IV and ST8Sia II sequences ordered by the percentage of conserved amino acid positions specific to ST8Sia IV (yellow background) and ST8Sia II (orange background) present in each sequence**. Five *B. floridae *ST8Sia II/IV sequences cannot be assigned to either ST8Sia II or ST8Sia IV subfamilies, because they contain the same proportion of ST8Sia II and ST8Sia IV specific amino acid positions (50 ± 9%). This intermediate position, with a relative large number of total ST8Sia II plus ST8Sia IV positions (28–31 out of 39 colored positions) suggests that these sequences are orthologues to the common ancestor, expected to be present before the duplication event responsible of the ST8Sia II and ST8Sia IV genes. The total number of subfamily-specific conserved amino acid positions is followed by the relative proportion (%) of ST8Sia II and ST8Sia IV specific positions for each protein. The first amino acid position of each line corresponds to the initial amino acid of sialylmotif L.

The second branch contained all the α2,8-sialyltransferases, also termed polysialyltransferases, which include the ST8Sia II and ST8Sia IV subfamilies and an additional branch containing the invertebrate sequences from *B. floridae *(Bfl-16 and Bfl-20). These *B. floridae *sequences may represent orthologs to the common ancestor of the two ST8Sia II and ST8Sia IV vertebrate subfamilies. From a comparison of the respective amino acid sequences, the invertebrate gene products could not be assigned to either the ST8Sia II or ST8Sia IV subfamilies because they were approximately equally divergent (41 and 59% conserved amino acid positions relative to ST8Sia II and ST8Sia IV subfamilies, respectively; Fig. 4). Interestingly, ST8Sia IV is not found in neognathi genomes, raising the possibility of a gene loss particular to these fish species [30] (additional file 2).

The third branch (Fig. 3) contained the vertebrate ST8Sia III and ST8Sia III-r subfamilies, as well as a group of invertebrate sequences from *B. floridae *(Bfl-12 and Bfl-17). These invertebrate sequences appear to be orthologues to the common ancestor of the subfamilies ST8Sia III and ST8Sia III-r that has disappeared in tetrapods. Within fish genomes, ST8Sia III-r is found only in the neognathi (*T. nigroviridis*, *T. rubripes*, *G. aculeatus*, *O. latipes*), but not in cyprinidae (*D. rerio*), nor in salmonidae (*O. mykiss*). It is interesting to note that the species devoid of the ST8Sia IV gene have the ST8Sia III-r gene (additional file 2).

The fourth branch grouped all the vertebrate α2,8-sialyltransferases subfamilies (ST8Sia I, ST8Sia V, ST8Sia VI), which catalyze the transfer of a single sialic acid residue and a new ST8Sia VII subfamily found in the fishes *D. rerio *and *O. mykiss *and the green lizard *Anolis carolinensis *(Fig. 3 and additional file 3). It was noted that the amphioxus gene product Bfl-9 branched out before the divergence of these vertebrate subfamilies suggesting that it could represent an ortholog to the common ancestor of the ST8Sia I, ST8Sia V, ST8Sia VI and ST8Sia VII subfamilies (Fig. 3). The ST8Sia VII subfamily is absent in neognathi (Fig. 3 and additional file 2).

### The ST8Sia subfamily motifs

**Figure 5:**
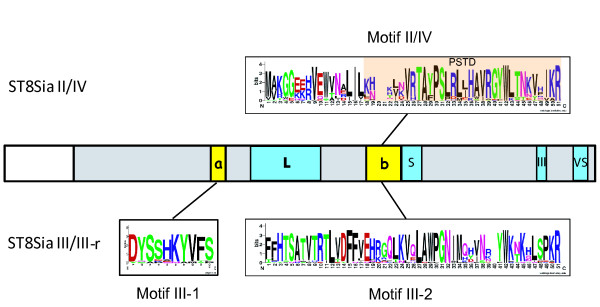
**Polysialyltransferase domain (PSTD) found in vertebrate ST8Sia II and ST8Sia IV and in invertebrate sequences and the two new motifs specific to the ST8Sia III subfamilies**. The catalytic domain of the sialyltransferases is indicated by the grey rectangles, the sialylmotifs L, S, III and VS by blue boxes and the a and b motifs by yellow boxes. The conserved peptide sequences used to generate the sequence logos of motif II/IV, motif III-1 and motif III-2 were extracted from multiple sequence alignments of vertebrate and invertebrate sequences. The overall height of the stack indicates the sequence conservation at a given position, while the height of symbols within the stack indicates the relative frequency of each amino acid at that position [69,70]. In the logos, amino acids are colored according to their chemical properties. The PSTD, which is included in the larger motif II/IV is shown with the orange background.

As previously described [14], the marine invertebrate ST8Sia amino acid sequences had intermediate values of subfamily-specific conserved amino acid positions in all the ClustalRO two by two alignments. In addition, they appeared at the roots of the ST8Sia II/ST8Sia IV branch, the ST8Sia III/ST8Sia III-r branch and the ST8Sia I/ST8Sia V/ST8Sia VI/ST8Sia VII branch in the phylogenetic tree (Fig. 3). We thus looked for peptide motifs that could uniquely represent each of these ST8Sia groups. We identified a specific motif characteristic of the ST8Sia II/ST8Sia IV group in the *B. floridae *sequences (Bfl-6, Bfl-14, Bfl-15, Bfl-16 and Bfl-20) (Fig. 4). This (b) motif located upstream of the sialylmotif S (Fig. 5) contains the polysialyltransferase domain (PSTD), a 32 amino acid sequence initially described by Nakata *et al. *[31] in the ST8Sia II and ST8Sia IV sequences. The polybasic PSTD motif appeared to be a functional motif involved in the elongation of linear chains of sialic acid. In addition, we identified a new specific peptide motif III-1 (a) located upstream of sialylmotif L and, a new specific peptide motif III-2 (b) located upstream of sialylmotif S in the amphioxus sequences Bfl-12 and Bfl-17 (Fig. 3), the sea urchin sequence Spu-7 (additional files 3 and 4) and in all the other members of the vertebrate ST8Sia III and ST8Sia III-r subfamilies. No peptide sequence motif specific for the mono-α2,8-sialyltransferases could be identified in the vertebrate ST8Sia I, ST8Sia V, ST8Sia VI and ST8Sia VII subfamilies, nor in the corresponding *B. floridae *and *S. purpuratus *sequences. Similarly, no distinguishing motif could be defined for the marine invertebrate sequences belonging to the ST8Sia EX group.

### Analysis of conserved gene synteny and orthology

**Figure 6:**
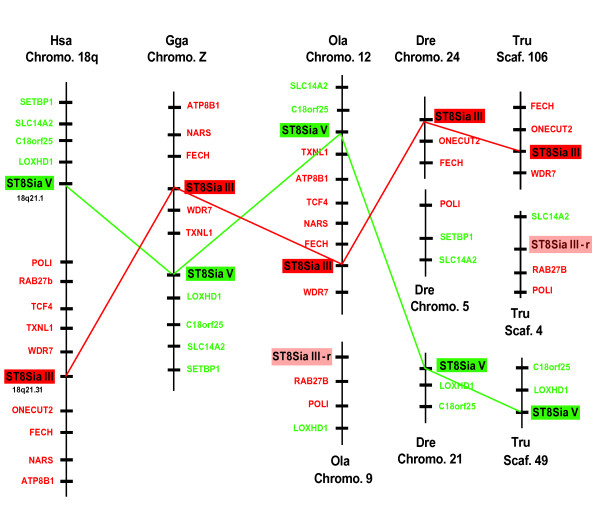
**Syntenic relationships of the ST8Sia III, ST8Sia III-r and ST8Sia V gene loci in fish and tetrapods (chromosomal locations of ST8Sia III, ST8Sia III-r and ST8Sia V in various vertebrate species)**. The physically-mapped genomes of human (Hsa), chicken (Gga), *Oryzias latipes *(Ola), *Danio rerio *(Dre) and *Takifugu rubripes *(Tru) were used to identify conserved gene neighbours of the ST8Sia III, ST8Sia III-r and ST8Sia V genes and to identify orthologs in fishes. Putative orthologies were determined with information available from the Ensembl server and by searching the various genomes using the ST8Sia protein sequences as TBLASTN queries. In each panel, each diagram represents the order of genes on the chromosome in the vicinity of the relevant ST8Sia gene. The names of the ST8Sia III neighbour genes are red and the ST8Sia III gene is a red box, whereas the names of the ST8Sia V neighbour genes are green and the ST8Sia V gene is a green box. The ST8Sia III-r gene is a pink box.

**Figure 7:**
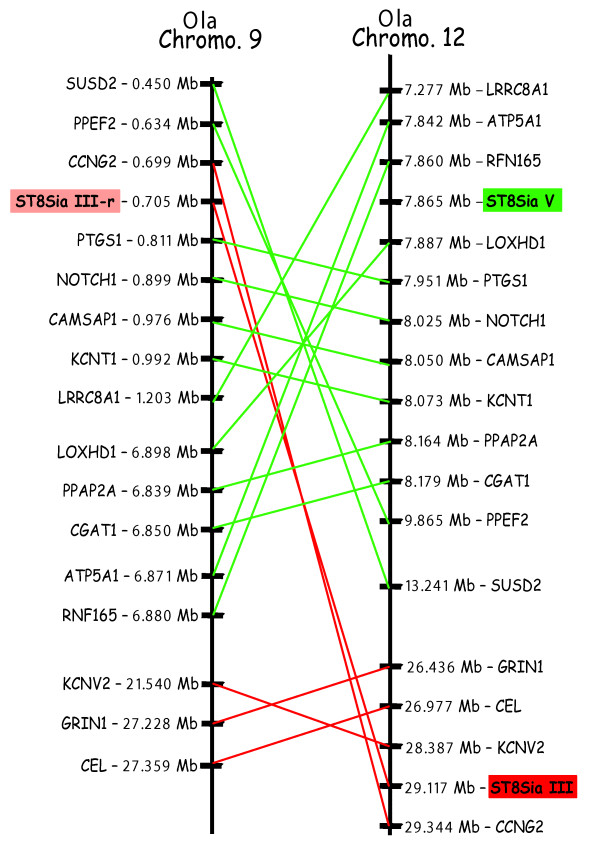
**Fish-specific paralogons in the medaka genome in the vicinity of the ST8Sia III and ST8Sia III-r genes**. The physically-mapped genome of medaka *O. latipes *(Ola) was used to identify conserved gene neighbours of the ST8Sia III and ST8Sia III-r genes and to identify orthologs by chromosomal walking and reciprocal TBLASTN searches of genes adjacent to ST8Sia loci. Putative orthologs were determined with information available from the Ensembl server. Paralogous ST8Sia III neighbour genes are linked with red lines and the ST8Sia III gene is indicated by a red box whereas ST8Sia V neighbour genes are linked with green lines and the ST8Sia V gene is shown as a green box. The ST8Sia III-r gene is denoted by a pink box.

In order to explain the appearance of the eight vertebrate ST8Sia subfamilies, we further analyzed the evolutionary history of ST8Sia in the context of the two rounds of whole genome duplications (known as the 2R hypothesis) [21], tandem duplication of genes, gene loss, synteny and datation. The same chromosomal location of ST8Sia III and ST8Sia V on human chromosome 18q is indicative of a tandem duplication that dates back at least to the osteichthyan emergence, since this tandem position is also found in chicken (*G. gallus*) and in medaka (*O. latipes*). There is an overall conservation of syntenic organization around these two genes in these three organisms. In *D. rerio *and in *T. rubripes*, the syntenic region is spread over three different chromosomes or scaffolds (Fig. 6). Moreover, two copies of ST8Sia III are included in two paralogons on *O. latipes *chromosomes 9 and 12 (Fig. 7).

**Figure 8:**
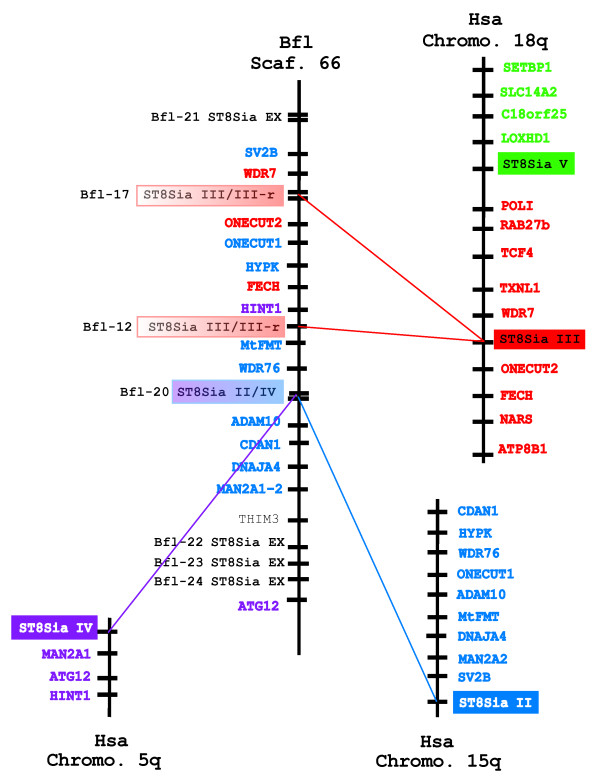
**Conserved synteny between a *Branchiostoma floridae *genomic region of scaffold 66 hosting the ST8Sia II/IV ST8Sia III/III-r and ST8Sia EX genes and human paralogons**. Synteny between the human ST8Sia gene loci and related genes on scaffold 66 of the marine invertebrate *B. floridae *(Bfl) were assessed by chromosomal walking and reciprocal TBLASTN searches of genes adjacent to ST8Sia loci in human (Hsa) and amphioxus (Bfl) genome databases. The names of the ST8Sia III neighbour genes are red and the ST8Sia III gene is represented by a red box. The names of the ST8Sia V neighbour genes are green and the ST8Sia V gene is indicated by a green box. The names of the ST8Sia II neighbour genes are blue and the ST8Sia II is shown as a blue box. The names of the ST8Sia IV neighbour genes are violet and the ST8Sia IV gene is denoted by a violet box. The ST8Sia III/III-r genes are white/red boxes and the ST8Sia II/IV gene is depicted by a violet/blue box.

Furthermore, scaffold 66 of *B. floridae *contains genes belonging to the ST8Sia EX group defined by phylogeny, orthologous sequences for ST8Sia II/IV (Bfl-20), for ST8Sia III/III-r (Bfl-12 and Bfl-17) and for their neighboring genes (Fig. 8). This denotes the common origin of these ST8Sia genes from an old conserved synteny and suggests a series of tandem duplications. However, scaffold 66 lacks the region around ST8Sia V. Finally, we found statistically significant human paralogons (sm>3) containing the ST8Sia II and ST8Sia IV genes described by McLysaght *et al. *[20,32] on chromosomes 15 (HSA15) and 5 (HSA5). These two ST8Sia genes share a conserved syntenic relationship from fish to mammals [33] and our results suggest that they might have arisen from a common invertebrate ancestor (Fig. 8).

### Time of gene duplication and evolutionary history of the ST8Sia family

In order to estimate the time of divergence of the vertebrate ST8Sia subfamilies, we reconstructed linearized trees for duplicate genes under the assumption of a molecular clock. The results are given in Table 1 and additional file 5. It appears that the equations fall into 3 groups. The first group contains the subfamilies ST8Sia II and ST8Sia IV, the second contains the subfamilies ST8Sia III and ST8Sia III-r, and the third contains the subfamilies ST8Sia I, ST8Sia V, ST8Sia VI and ST8Sia VII. A comparison of the regression slopes between pairs of groups reveals that the ST8Sia I/ST8Sia V/ST8Sia VI/ST8Sia VII has a significantly lower slope than that of the ST8Sia III/ST8Sia III-r group (F = 6.74, p < 0.01) and slightly lower to that of ST8Sia II/ST8Sia IV (F = 3.01, p = 0.08). However, branch-site test does not show a positive selection in any ST8Sia subfamily, even in the ST8Sia VI group, where the highest evolution rate is recorded (Table 2). The earliest node datations refer to the duplications between ST8Sia I and ST8Sia V/ST8Sia VI/ST8Sia VII and between ST8Sia V and ST8Sia VI/ST8Sia VII, around 596 and 563 MYA respectively (Table 3). We estimate that the duplications between ST8Sia II and ST8Sia IV and between ST8Sia VI and ST8Sia VII took place about 552 MYA. The latest duplication event was estimated to be around 474 MYA and corresponds to the divergence between ST8Sia III and ST8Sia III-r.

**Table 1:** Regression equations of linearized distances versus million years ago (MYA).

ST8Sia group	Regression equations	Determination coefficient	Significance
ST8Sia II/ST8Sia IV	y = 920x + 296	R^2 ^= 0,90	p = 0.00085 (eq1)
ST8Sia III/ST8Sia III-r	y = 752x + 326	R^2 ^= 0,90	p = 0.025 (eq2)
ST8Sia I/ST8Sia V/ST8Sia VI/ST8Sia VII	y = 430x + 313	R^2 ^= 0.88	P < 0.001 (eq3)

**Table 2:** Estimation of the number of synonymous (*d*_S_) and nonsynonymous (*d*_N_) substitutions per site for ST8Sia genes by the branch-site model (PAML version 4.0).

Subfamily	κ	*d*_N_(i)	*d*_S_(i)	ω **(i)**	ω **(b)**	ω **(i)/ω (b)**
ST8Sia I	2.204	0.176	0.235	0.748	0.495	1.512
ST8Sia II	2.201	0.052	0.133	0.387	0.556	0.696
ST8Sia III	2.202	0.225	0.433	0.518	0.529	0.980
ST8Sia IV	2.205	0.044	0.170	0.258	0.593	0.435
ST8Sia V	2.202	0.163	0.337	0.485	0.535	0.907
ST8Sia VI	2.205	0.045	0.054	0.792	0.483	1.638

For the 31 genes belonging to the different subfamilies, 271 codons have been taken into account; κ corresponds to estimated transition over transversion ratio and ω to *d*_N_/*d*_S_; (i) branches of interest, (b) background branches.

**Table 3:** The inferred datation of each node

Nodes	Time in MYA [Conf. interv. 95%]	Equation used
ST8Sia I – ST8Sia V/ST8Sia VI/ST8Sia VII	596 [544–647]	eq (3)
ST8Sia V – ST8Sia VI/ST8Sia VII	563 [512–614]	eq (3)
ST8Sia II – ST8Sia IV	552 [598–506]	eq (1)
ST8Sia VI – ST8Sia VII	551 [500–602]	eq (3)
ST8Sia III – ST8Sia III-r	474 [541–407]	eq (2)

## Discussion

### Model of divergent evolution with punctual areas of gene loss and birth

**Figure 9:**
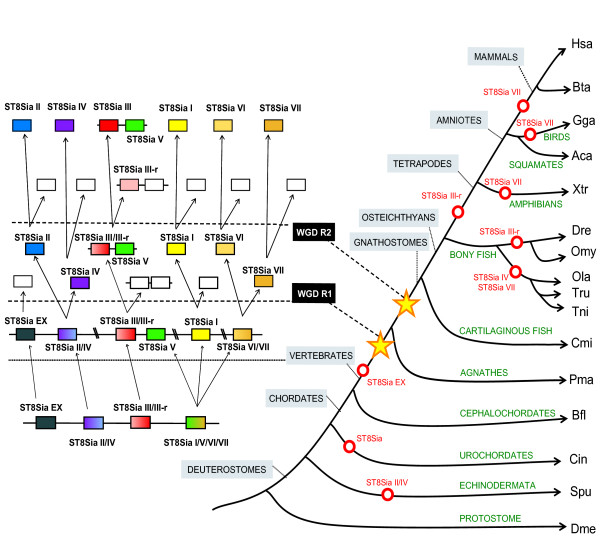
**Schematic drawing of the evolution of the ST8Sia gene family**. Model for the evolution of the ST8Sia genes based on evidence from protein sequence phylogeny, conserved synteny of genomic loci between species and paralogous relationships between the genomic regions of human ST8Sia. The diagram takes into account that the four ancestral groups of ST8Sia (four bottom coloured boxes) predate the WGD R1 and WGD R2, which occurred early in the vertebrate lineage. Dotted lines are intermediate steps. Open red circles are gene losses on the phylogenetic tree. Open boxes represent gene loss following block duplications. The broken lines correspond to the two main WGDs R1 and R2.

Our investigations have established an orthologous relationship between the mammalian ST8Sia genes and their invertebrate deuterostome counterparts. To date, ST8Sia genes have been identified only in the deuterostome lineage, and no ST8Sia sequences have been identified in the roundworm *C. elegans *nor in insects such as honey bee, mosquito or fruit fly [14]. The current paradigm of WGD predicts a first round of duplication after the emergence of vertebrates but before the separation between agnathans and gnathostomes, followed by a second round of duplication after this divergence [34]. In figure 9, we propose a scenario that illustrates how the ST8Sia gene family might have evolved, that is consistent with their phylogenetic relationships, with synteny, with the paralogon analysis, and tandem duplication or WGD and gene loss [35]. Since genes within the ST8Sia EX, ST8Sia II/IV and ST8Sia III/III-r groups are located on the same scaffold 66 of amphioxus (*B. floridae*) (Fig. 8), we hypothesize that they resulted from tandem duplications, which occurred before the separation of cephalochordates and vertebrates. The ST8Sia III and ST8Sia V genes are on the same chromosome from fish to humans indicating that they originated from a second ancient tandem duplication (Fig. 6). Phylogenetic analyses revealed that the duplication order is ST8Sia EX > ST8Sia II/ST8Sia IV > ST8Sia III/ST8Sia III-r > ST8Sia I/ST8Sia V/ST8Sia VI/ST8Sia VII. Within this last group, successive duplications took place early in the vertebrate lineage leading to the emergence of ST8Sia I, ST8Sia V, ST8Sia VI and ST8Sia VII. Based on the present vertebrate genome data, the chromosomal segment bearing the ancestor of ST8Sia II/ST8Sia IV, ST8Sia III/ST8Sia III-r, ST8Sia VI/ST8Sia VII and ST8Sia I migrated to four different chromosomes early in the differentiation of the vertebrate phylum (Fig. 9). Unequivocal paralogons including the ST8Sia II and ST8Sia IV genes were found between the human chromosome 5 and 15 (HSA 5 and HSA 15) [20] and we identified an ortholog to ST8Sia IV in the lamprey (*P. marinus*) suggesting that these genes result from a block duplication that occurred before the divergence between gnathostomes and agnathans. The association of this event with the first round of WGD is supported by the datation of around 552 MYA, close to WGD R1 [36]. The duplication event within the ST8Sia VI/ST8Sia VII group dates back around 551 MYA, but unfortunately, no paralogon around these genes could be identified making it difficult to draw conclusions regarding the mechanism involved in the emergence of both genes. Paralogons observed in medaka (Fig. 7) and topology of the phylogenetic tree shown in Fig. 3 indicates that ST8Sia III and ST8Sia III-r genes result from a block duplication, probably related to WGD R2 (datation around 474 MYA, close to R2) [36], and not to the more recent WGD R3 (genome duplication specific to teleosts), around 320 MYA [37]. The two rounds of WGD should have generated twenty genes, but only eight of them remained in the living vertebrate species. Notably, species specific tandem duplications have resulted in duplicated genes: (i) ST8Sia VIIA and ST8Sia VIIB in *D. rerio *and *A. carolinensis*, that are organized head to tail on the same chromosome (ii) ST8Sia VIA and ST8Sia VIB in *T. rubripes *and (iii) ST8Sia IIA and ST8Sia IIB genes in *O. mykiss *[38]. Considering the order of the successive duplications and the presence of genes orthologous to ST8Sia EX, ST8Sia III/ST8Sia III-r and ST8Sia I/ST8Sia V/ST8Sia VI/ST8Sia VII in the sea urchin (*S. purpuratus*), we deduced that these duplications date back to the common ancestor of sea urchin and vertebrates, early in the deuterostome lineage. In addition, the absence of an ortholog to ST8Sia II/ST8Sia IV in *S. purpuratus *might result from a gene loss. Similarly, the absence of the ST8Sia EX counterparts in vertebrates is certainly due to a gene loss in their common ancestor (Fig. 9). Moreover, the absence of any ST8Sia gene in the tunicate genomes (*C. intestinalis *and *C. savignyi*) can be explained in the context of their probable ancestral colinearity by a single deletion of the ST8Sia cluster. In summary, a series of ancient gene duplications and the two WGDs account for much of the ST8Sia gene diversity in vertebrates. In contrast, the expansion and subsequent divergence of the vertebrate fucosyltransferase genes appears to primarily be the consequence of WGD R1 and R2 [39].

### Diversification of functions

The vertebrate enzymes of the ST8Sia family catalyze the transfer of sialic acid in an α2,8-linkage to other sialic acid residues present in glycoproteins and glycolipids [15]. We describe here for the first time the ST8Sia EX family, a new group of genes restricted to the non-vertebrate marine deuterostomians Bfl and Spu. These putative gene products possess all the characteristic peptide motifs of the ST8Sia family [13], including the new C-term motif, suggesting that they might have α2,8-sialyltransferase activity. The presence in sea urchin of novel polysialylated structures such as α2,9-linked polysialic acid chains [40] or (5-O-glycolyl-Neu5Gcα2-)_n _sequences, where n ranges from 4 to more than 40 [41-43] raises the question of the role played by ST8Sia EX gene products in their biosynthetic pathway. The region in the ST8Sia EX amino acid sequences corresponding to the PSTD motif found in the polysialyltransferases ST8Sia II/ST8Sia IV is quite different from the consensus PSTD motif, suggesting that these enzymes might carry out a different form of sialylation (i.e. α2,9-sialylation). In addition, α2,9-linked polysialylated structures were also described in mouse neuroblastoma cells [44], but no ortholog to ST8Sia EX could be identified in the mouse genome suggesting that the ST8Sia EX genes might have evolved in invertebrate deuterostomes to achieve a novel sialylation.

The ST8Sia II/ST8Sia IV group characterized by a PSTD is found from cephalochordates to mammals. In vertebrates, ST8Sia II and ST8Sia IV can assemble long, linear polysialic acid chains (50–200 residues) on the *N*-glycans of the neural cell adhesion molecule N-CAM and also on the polysialoglycoprotein (PSGP) in fish eggs [45,46]. In spite of the lack of data on the presence of α2,8-sialylation in the glycoconjugates of amphioxus, we predict that these polysialylated structures exist because several genes of *B. floridae *code for putative proteins possessing the PSTD-like motif. Yet, no such polysialylated structure has been found in sea urchin (*S. purpuratus*) glycoconjugates, a fact correlated with the absence of ST8Sia II/ST8Sia IV genes in this genome. However, an α2,8-polysialyltransferase activity has been demonstrated in another developmentally regulated sea urchin species (*Lytechinus pictus*), with a peak at the gastrula stage [47]. In this species, the enzyme activity is probably associated with the migration or movements of cells during gastrulation. This is comparable with the role that polysialylation serves in increasing neuronal plasticity and migration in embryonic vertebrates [33], through the modification of N-CAM. Two enzymes, ST8Sia II and ST8Sia IV that carry out the polysialylation of the *N*-glycans of N-CAM are present in most vertebrates. For example, the temporal pattern of expression of the ST8Sia II gene is restricted to the early development stages. In contrast, ST8Sia IV is expressed at lower levels from the later stages of development to adulthood [33]. This can be related to a weaker selective pressure on ST8Sia IV than on ST8Sia II, as illustrated by longer branch lengths corresponding to higher mutation rates (additional file 5).

**Figure 10:**
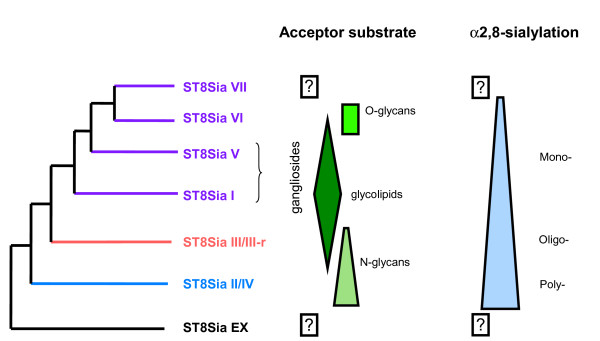
**Schematic drawing of the evolution of sialylation with a general trend from poly- to oligo- and mono-α2,8-sialylation patterns**. Acceptor substrates and mode of sialylation are given on the left side of the simplified phylogenetic tree. The boxed question mark indicates that the enzymatic activities of ST8Sia EX and ST8Sia VII are unknown.

Mammalian ST8Sia III was shown to add α2,8-linked sialic acid to terminal α2,3-linked sialic acid of glycolipids and glycoproteins, resulting in mono- to oligo-α2,8-sialylation of *N*-glycans of glycoproteins (Fig. 10), with the notable exception of those of N-CAM [48,49]. In addition, the human enzyme catalyzes the synthesis of short sialyl-oligomers (<10 residues) suggesting that ST8Sia III is an oligosialyltransferase [48,50]. The protein sequences of the ST8Sia III/ST8Sia III-r group show the same evolutionary rate as those of the ST8Sia II/ST8Sia IV group. In zebrafish, the ST8Sia III gene is mainly expressed in the 36 h post fertilization embryo with a lower of expression in the adult brain (Fig. 2). It also exhibits a restricted expression in mouse, notably in brain and testes [51]. Interestingly, up to date, we described the ST8Sia III-r gene in the neognathi bony fishes, animal species in which the ST8Sia IV gene is missing, which suggests that ST8Sia III-r might replace ST8Sia IV activity (additional file 2). It can be excluded that both subfamilies are orthologous given the nearly 100% bootstrap value associated to the branch common to ST8Sia III and ST8Sia III-r.

Phylogenetic analysis indicates that the genes of the ST8Sia I/ST8Sia V/ST8Sia VI/ST8Sia VII group evolved faster than the other ST8Sia genes because they form longer branches. Among them, ST8Sia VI has the highest mutation rate, not associated with positive selection as indicated by a *d*_N_/*d*_S _ratio < 1 (Table 2) and it shows a low level of basal expression in zebrafish tissues (Fig. 2). The mammalian ST8Sia VI transfers only one sialic acid residue and synthesizes disialylated structures on *O*-glycans [52,53]. Interestingly, the expression of the ST8Sia VII gene is restricted to a subset of non-mammalian vertebrates, which include the lamprey (*P. marinus*), teleosteans (*D. rerio *and *O. mykiss*), and the green lizard (*A. carolinensis*) and it exhibits remarkable tissue specific expression pattern in zebrafish, primarily in the ovary and intestine (Fig. 2). The enzymatic activity of this enzyme is not known, but we hypothesize that it could be a mono-α2,8-sialyltransferase responsible for the particular disialylated structures (NeuAc/NeuGc) recently described in *D. rerio *[4]. In this gene group, the two last members ST8Sia I and ST8Sia V have the same expression profile in zebrafish (Fig. 2). Both enzymes are involved in ganglioside biosynthesis. ST8Sia I transfers one sialic acid residue to the α2,3-linked sialic acid residue of GM3 to make GD3 [54-56] whereas ST8Sia V synthesizes GD1c, GT1a, GQ1b and GT3 [57].

## Conclusion

The genomic analysis we have presented gives new insights into the events leading to a birth and death model for the evolution of the genes encoding α2,8-sialyltransferases. Much of the diversity is due to gene duplication events occurring early in the deuterostome lineage, most likely rapidly after the emergence of an ancestral ST8Sia gene. Based on results from this study we propose that the newly identified, novel ST8Sia EX gene group in the invertebrate genomes is a candidate ancestral ST8Sia gene. The assembly of polysialic acid glycans on glycoconjugates took place very early in animal evolution (Fig. 10), with the emergence of the ST8Sia II/ST8Sia IV group early in the evolution of deuterostomes (~ 750 MYA) followed by the emergence of the ST8Sia III/ST8Sia III-r group. The relative conservation of the PSTD-like motif in these latter sequences (motif III-2, Fig. 5) and the fact that ST8Sia III may drive oligosialylation of glycoconjugates, further suggests that ST8Sia III-r might also be involved in oligosialylation (Fig. 10). In contrast, the group of mono α2,8-sialyltransferases (ST8Sia I/ST8Sia V/ST8Sia VI/ST8Sia VII) underwent significant modifications in the PSTD-like motif. The rapid mutation rate throughout these vertebrate sequences led to novel α2,8-sialylation changes with respect to the length of the α2,8-sialic acid chains assembled and their association with specific acceptor substrates, including *O*-glycans (Fig. 10).

## Methods

### Sequences

Only eukaryote sequences were considered for this study. Orthologous ST8Sia proteins were identified from all genomic and EST sequences available in the general databanks such as NCBI for the green lizard *Anolis carolinensis*, [58], ENSEMBL [59] or DDBJ [60] or in specialized databases [61], JGI for the amphioxus *Branchiostoma floridae *[62], the Genome Sequencing Center at the Washington University School of Medicine, St Louis MO for the sea lamprey *Petromyzon marinus *[63], the Genome Sequencing Center at the Baylor College of Medicine for *Homo sapiens *and the sea urchin *Strongylocentrotus purpuratus *[64], the Institute of Molecular and Cell Biology for the elephant shark *Callorhinchus milii *[65] using BLASTN, TBLASTN and PSI-BLAST [66] with default parameters (an *e*-value cut off at 0.01 was used in all BLAST searches). Human and mouse sequences were used as first queries in the first round of search. Contigs of the different ESTs of each gene were made with CAP3 [67]. New complete open reading frames identified in these EST-CAP searches with more than two identical amino acids overlapping in each position were annotated and submitted to EMBL/GenBank as putative ST8Sia sequences. All genomic sequences allowing generation of a complete catalytic domain were considered. Splice site prediction analysis was achieved at the Berkeley drosophila genome project.

### RT-PCR

*Danio rerio *ST8Sia sequences identified *in silico *were amplified by PCR with specific primers to determine expression. Total RNA was extracted from various zebrafish adult tissues using the Qiagen RNeasy kit, and RNA was quantified by spectrophotometry using the NanoDrop^® ^ND-1000 spectrophotometer (NanoDrop Technologies, Wilmington, DE, U.S.A.). In addition, the integrity and purity of the extracted RNA was analyzed by gel electrophoresis on a bioanalyzer (Experion, Bio-Rad Laboratories, Inc). For subsequent PCR amplifications, first-strand cDNA was synthesized from total RNA using the First Strand cDNA Synthesis kit according to the manufacturer's protocol (Amersham Pharmacia Biotech, Little Chalfont, U.K.) in the presence of oligodeoxythymidilic acid_12–18 _in a final volume of 33 μl. A specific fragment of about 370 bp was obtained from cDNA generated from different adult tissues using 35 nM of specific sense and antisense primers synthesized by Eurogentec, (Table 4), 100 μM of dNTP and 0.5 unit of Q-Biogen DNA Taq polymerase using the following protocol: 96°C for 2 min, 38 cycles of 45 sec at 95°C, 1 min at 50°C and 1 min at 72°C, and 10 min at 72°C. The same RT-PCR conditions using zebrafish β-actin were used as the control for cDNA synthesis and purity. The RT-PCR products were subjected to 2% agarose gel electrophoresis and amplification of cDNA resulted in a 378 bp fragment. All vertebrate and invertebrate sequences newly identified in this study and their accession numbers are listed in additional file 1.

**Table 4:** Primer nucleotide sequence and expected amplicon size. Accession numbers in GeneBank for the identified sialyltransferases and β-actin sequences.

Primer	Sequence	Accession number	Product size (bp)
ST8Sia I	Forward 5'-TTGCGGTTACTAAGGAGA Reverse 5'-ACGAAAGATTTGCGGGAC	AJ715535	346
ST8Sia II	Forward 5'-GACTCGCACGACTTTGTT Reverse 5'-TGGTTGGTCAGCCAGTAA	AY055462	335
ST8Sia III	Forward 5'-AACAACCTGCTGACCATCC Reverse 5'-ATGATACGGCAGCTCCTT	AJ715543	354
ST8Sia IV	Forward 5'-TCTTGACTTGGGAGTTGG Reverse 5'-TCTGACCGCAATCCTACA	AJ715545	366
ST8Sia V	Forward 5'-AAATAAGGAGGAGACGGATAA Reverse 5'-AAAGTCAGAAGCGTCAAT	AJ715546	291
ST8Sia VI	Forward 5'-TGTCTATGATGGCGAAAG Reverse 5'-TGACCGTATGAATGAAGG	AJ715551	333
ST8Sia VIIA	Forward 5'-TTTCCTGGTGGTCCTGAT Reverse 5'-GGTGCGTCTACTGTTGGTT	AM287257	346
β-actin	Forward 5'-GTTGGTATGGGACAGAAAGA Reverse 5'-GGCGTAACCCTCGTAGAT	AF025305	378

The transmembrane domain was determined using the TMPRED program available from the ExPASy proteomics server. Multiple sequence alignments were performed with ClustalW [68] at PBIL and EBI. Sequence logos were created using WebLogo (version 2.8.2; [69,70]).

### ClustalRO and multiple sequence alignments

The subfamily of each hypothetical sialyltransferase was further confirmed by determining the relative proportions of subfamily-specific conserved positions in ClustalRO two by two alignments as previously described [14]. This simple method, based on the similarities between sequences is complementary to the more sophisticated phylogeny calculations that are based on the differences between sequences.

### Phylogenetic analysis

The informative positions within protein alignments were selected by G-BLOCKS [29,71]. Maximum likelihood (ML) analyses were done with PhyML, version 2.4.4 [72] using the JTT model of amino-acid substitution. Bootstrap values for the nodes were determined by analyzing 500 replicates. The topology obtained from maximum likelihood was taken in the user tree option. To draw the trees, the generated nexus topology files were read by MEGA3.1 [73].

### Synteny analysis and paralogon detection

Synteny between vertebrate ST8Sia and related genes in invertebrates was assessed by chromosomal walking and reciprocal BLAST searches of genes adjacent to ST8Sia loci in the human (Hsa), mouse (Mmu), chicken (Gga) medaka (Ola), zebrafish (Dre), *T. rubripes *(Tru) and amphioxus (Bfl) genome databases (Ensembl). The identification of paralogous blocks [20] was done using the latest Ensembl dataset (version 5.28). The website for these paralogons [32] offers the possibility to carry out block detection in humans with self-defined parameters.

### Time of gene duplication/evolution rate

We recorded the branch lengths in the maximum likelihood tree linearized by Mega3.1for the following calibrations: sea urchin/vertebrates: 750 MYA [74]; amphioxus/vertebrates: 650 MYA [75]; lamprey/gnathostomes: 575 MYA; gnathostomes/osteichthyans: 460 MYA; osteichthyans/other vertebrates: 450 MYA; tetrapodes/actinopterygians: 360 MYA; amniotes/other vertebrates: 310 MYA; Genome duplication in teleosteans: 320 MYA [37]. We calculated the regression equations between linearized branch lengths and calibration dates by considering separately the 3 groups of subfamilies identified from phylogenetic analyses: ST8Sia II/ST8Sia IV, ST8Sia III/ST8Sia III-r and ST8Sia I/ST8Sia V/ST8Sia VI/ST8Sia VII. Confidence intervals at 95% were calculated as 1.96 times the standard deviations of regression equation residues. Comparison of regression slopes was performed with PAST [76]. We tested for evidence of positive selection using the branch-site method implemented in PAML version 4 [77], as previously described [78-80]. Briefly, we calculated the ratio of the nonsynonymous substitution rate (*d*_N_) to the synonymous substitution rate (*d*_S_) for each ST8Sia subfamily, in the branch of interest (ω (i) for one ST8Sia subfamily) and in the background branches (ω (b) for the remaining ST8Sia subfamilies). Thirty-one vertebrate ST8Sia sequences from *D. rerio*, *X. tropicalis*, *G. gallus *and *H. sapiens *were aligned in multiple sequence alignments with the exception of the ST8Sia VII sequences, which have not been identified in all these vertebrate species and 813 informative sites were G-BLOCKS selected. A user tree topology identical to the one described in figure 3 was obtained by Minimum Evolution option in MEGA 3.1 [73].

## Abbreviations

Hsa: *Homo sapiens*; Bta: *Bos taurus*; Mam: *Maccaca mulata*; Pma: *Petromyzon marinus*; Dre: *Danio rerio*; Aca: *Anolis carolinensis*; Ptr: *Pan troglodytes*; Mmu: *Mus musculus*; Rno: *Rattus norvegicus*; Cfa: *Canis familiaris*; Gga: *Gallus gallus*; Xtr: *Xenopus tropicalis*; Xla: *Xenopus laevis*; Omy: *Oncorhynchus mykiss*; Gac: *Gasterosteus aculeatus*; Tni: *Tetraodon nigroviridis*; Tru: *Takifugu rubripes*; Ola: *Orysias latipes*; Cmi: *Callorhinchus milii*; Bfl: *Branchiostoma floridae*; Spu: *Strongylocentrotus purpuratus*; Cin: *Ciona intestinalis*; Csa: *Ciona savignyi*; Cel: *Caenorhabditis elegans*; ST8Sia: α2,8-sialyltransferase nomenclature according to Tsuji *et al. *[81]; ST8Sia III-r: ST8Sia III-related; ST8Sia EX: ST8Sia external group; 2R: two rounds of whole genome duplication; MYA: million years ago; WGD: whole genome duplication. Gangliosides GM3, GD3, GD1c, GT1a, GQ1b and GT3 are named according to Svennerholm [82].

## Authors' contributions

AHL and RO conceived the study, AHL, DP, RO, JMP collected and analyzed the data, AHL, DP and RO wrote the paper, PD, RM, JMP provided substantial editorial advice, AHL and PD provided critical insights into ST biochemistry and biology and all authors have read and approved the final manuscript.

## Supplementary Material

Additional file 1 **Accession numbers of the newly identified ST8Sia**. Thirty five vertebrate and twenty seven invertebrate sequences were identified in databases. Click here for file

Additional file 2 **ST8Sia of ray-finned fish**. Accession numbers in GeneBank/EMBL of the sialyltransferases identified in fish and in human. The pale pink background indicates the duplicated genes identified in fish and squamates. ST8Sia IV could not be identified (not id., pale green background) in the neognathi fish and ST8Sia VII was identified only in the cyprinidae and salmonidae fish and in the green lizard. Click here for file

Additional file 3 **Phylogenetic ML tree (PhyML software) with 129 sequences**. One hundred and seventy three sites out of 521 positions (33%) within 10 informative blocks were identified with G-BLOCKS. Maximum Likelihood phylogenetic tree was constructed with Phyml, JTT model of amino acid substitution and 500 replicates. The mono-α2,8-sialyltransferases are yellow, the oligo-α2,8-sialyltransferases are green, the poly-α2,8-sialyltransferases are blue and the invertebrate ST8Sia EX sequences are pink. Click here for file

Additional file 4 **Phylogenetic ML tree (PhyML software) with 12 human sialyltransferases and all the invertebrates ST8Sia sequences**. One hundred and six sites out of 550 positions (19%) were selected with G-BLOCKS to find the most phylogenetically basal group within ST8Sia. User tree: topology obtained from Max likelihood with 500 replicates. The human ST6GalNAc III (AJ507291), ST6GalNAc IV (AJ271734), ST6Gal I (X17247), ST6Gal II (AB059555), ST3Gal I (L29555), ST3Gal IV (L23767) and the six human ST8Sia (ST8Sia I to ST8Sia VI) are indicated in green. All the invertebrate ST8Sia EX sequences are in a single red vanishing box. Click here for file

Additional file 5 **Linear regression curves calculated for each group of sialyltransferases**. **A**: poly-α2,8-sialyltransferase group (ST8Sia II and ST8Sia IV); **B**: oligo-α2,8-sialyltransferase group (ST8Sia III and ST8Sia III-r); **C**: mono-α2,8-sialyltransferases group (ST8Sia I, ST8Sia V, ST8Sia VI and ST8Sia VII). The equation of regression curves between the linearized branch lengths and datations in MYA are indicated in open boxes. Green diamonds refer to ST8Sia IV in graph A, and to ST8Sia VI in graph C. Click here for file
