# Supplementary material for: Evolutionary history of the alpha2,8-sialyltransferase (ST8Sia) gene family: Tandem duplications in early deuterostomes explain most of the diversity found in the vertebrate ST8Sia genes
Source: BMC Evol Biol. 2008 Sep 23;8:258. doi: 10.1186/1471-2148-8-258 (PMC2564942; doi:10.1186/1471-2148-8-258)
Supplement: Additional file 1 — Accession numbers of the newly identified ST8Sia. Thirty five vertebrate and twenty seven invertebrate sequences were identified in databases. [file 1471-2148-8-258-S1.pdf]

Additional file 1. **Newly identified sialyltransferases-related sequences.** 35 vertebrate and 27 invertebrate sequences were identified in the databases.

DNA GenBank/EBI	Vertebrates species	Subfamily	Predicted ORF size (amino acid)
AM422142	*G. aculeatus*	ST8Sia I	335
AM494543	*C. milii*	ST8Sia I	351
AM503578	*A. carolinensis*	ST8Sia I	>337
AM900683	*P. marinus*	ST8Sia I	>286
AM422134	*O. latipes*	ST8Sia II	372
AM422132	*C. familiaris*	ST8Sia II	332
AM422133	*B. taurus*	ST8Sia II	366
AM422139	*G. aculeatus*	ST8Sia II	375
AM503579	*A. carolinensis*	ST8Sia II	366
AJ715544	*S. tropicalis*	ST8Sia III	375
AM422140	*G. aculeatus*	ST8Sia III	374
AM422141	*G. aculeatus*	ST8Sia III-r	373
AM503580	*A. carolinensis*	ST8Sia III	381
AM419014	*S. tropicalis*	ST8Sia IV	359
AM419015	*R. norvegicus*	ST8Sia IV	455
AM503581	*A. carolinensis*	ST8Sia IV	360
AM900682	*P. marinus*	ST8Sia IV	>277
AM903381	*C. milii*	ST8Sia IV	301
AM287263	*D. rerio*	ST8Sia V	411
AM491803	*C. familiaris*	ST8Sia V	>363
AM491802	*M. mulatta*	ST8Sia V	376
AM422136	*S. tropicalis*	ST8Sia V	374
AM422135	*G. aculeatus*	ST8Sia V	379
AM494542	*C. milii*	ST8Sia V	372
AM503582	*A. carolinensis*	ST8Sia V	376
AM422137	*S. tropicalis*	ST8Sia VI	387
AM419017	*G. aculeatus*	ST8Sia VI	358
AM419016	*T. nigroviridis*	ST8Sia VI	337
AM422138	*O. latipes*	ST8Sia VI	358
AM503583	*A. carolinensis*	ST8Sia VI	>300
AM287257	*D. rerio A*	ST8Sia VIIA	358
AM287258	*D. rerio B*	ST8Sia VIIB	363
AM503584	*A. carolinensis A*	ST8Sia VIIA	>268
AM503585	*A. carolinensis B*	ST8Sia VIIB	>273
AM900684	*P. marinus*	ST8Sia VII	>275

DNA GenBank/EBI	Invertebrates species	Group	Predicted ORF size (amino acid)
EF126286	*S. purpuratus 1*	ST8Sia I/V/VI/VII	264
EF126287	*S. purpuratus 2*	ST8Sia I/V/VI/VII	323
EF126289	*S. purpuratus 4*	ST8Sia I/V/VI/VII	305
EF126290	*S. purpuratus 5*	ST8Sia I/V/VI/VII	352
EF126291	*S. purpuratus 6*	ST8Sia I/V/VI/VII	346
EF126293	*S. purpuratus 8*	ST8Sia I/V/VI/VII	304
EF134719	*S. purpuratus 9*	ST8Sia I/V/VI/VII	317
EF134720	*S. purpuratus 10*	ST8Sia I/V/VI/VII	323
EF156412	*B. floridae 4*	ST8Sia I/V/VI/VII	281
EF152419	*B. floridae 8*	ST8Sia I/V/VI/VII	267
EF152420	*B. floridae 9*	ST8Sia I/V/VI/VII	271
EF158036	*B. floridae 11*	ST8Sia I/V/VI/VII	291
EF126292	*S. purpuratus 7*	ST8Sia III/III-r	335
AM901543	*B. floridae 17*	ST8Sia III/III-r	292
AM901542	*B. floridae 12*	ST8Sia III/III-r	311
EF134720	*S. purpuratus 11*	ST8Sia EX	286
AF391289	*B. floridae 0*	ST8Sia EX	363
EF134722	*B. floridae 1*	ST8Sia EX	318
EF134723	*B. floridae 2*	ST8Sia EX	327
EF140743	*B. floridae 3*	ST8Sia EX	295
EF140744	*B. floridae 5*	ST8Sia EX	287
EF141188	*B. floridae 7*	ST8Sia EX	271
EF140745	*B. floridae 6*	ST8Sia II/IV	328
AM901544	*B. floridae 14*	ST8Sia II/IV	287
AM901545	*B. floridae 15*	ST8Sia II/IV	278
AM901546	*B. floridae 16*	ST8Sia II/IV	279
AM901547	*B. floridae 20*	ST8Sia II/IV	289
