# Supplementary material for: Evolutionary history of the alpha2,8-sialyltransferase (ST8Sia) gene family: Tandem duplications in early deuterostomes explain most of the diversity found in the vertebrate ST8Sia genes
Source: BMC Evol Biol. 2008 Sep 23;8:258. doi: 10.1186/1471-2148-8-258 (PMC2564942; doi:10.1186/1471-2148-8-258)
Supplement: Additional file 2 — ST8Sia of ray-finned fish. Accession numbers in GeneBank/EMBL of the sialyltransferases identified in fish and in human. The pale pink background indicates the duplicated genes identified in fish and squamates. ST8Sia IV could not be identified (not id., pale green background) in the neognathi fish and ST8Sia VII was identified only in the cyprinidae and salmonidae fish and in the green lizard. [file 1471-2148-8-258-S2.pdf]

Additional file 2: **ST8Sia family in ray-finned fish:** Accession numbers in GeneBank/EMBL are indicated for sialyltransferases identified in fish and in human. The pale pink background indicates the duplicated genes identified in fish and squamates. ST8Sia IV could not be identified (not id., pale green background) in the neognathi fish and ST8Sia VII was identified only in the cyprinidae and salmonidae fish and in the green lizard.

			**ST8Sia I**	**ST8Sia II**	**ST8Sia III**	**ST8Sia III-r**	**ST8Sia IV**	**ST8Sia V**	**ST8Sia VI**	**ST8Sia VII**
**Mammals**	**Hominidae**	*H. sapiens*	D26360	U33551	AF004668	Not id.	L41680	U91641	AJ621583	Not id.
**Squamates**	**Iguanidae**	*A. carolinensis*	AM503578	AM503579	AM503580	Not id.	AM503581	AM503582	AM503583	AM503584<br>AM503585
**Ray-finned fish**	**Cyprinidae**	*D. rerio*	AJ715535	AY055462	AJ715543	Not id.	AJ715545	AJ715546	AJ715551	AM287257<br>AM287258
	**Neognathi**	*T. nigroviridis*	AJ715536	AJ715537	AJ715539	AJ715540	Not id.	AJ715548	AM419016	Not id.
		T. rubripes	AJ715534	AJ715538	AJ715541	AJ715542	Not id.	AJ715547	AJ715549<br>AJ715550	Not id.
		G. aculeatus	AM422142	AM422139	AM422140	AM422141	Not id.	AM422135	AM419017	Not id.
		O. latipes	AJ871606	AM422134	AJ871607	AJ871608	Not id.	AJ871609	AM422138	Not id.
	**Salmonidae**	*O. mykiss*	partial	AB262976<br>AB262977	AB262974	Not id.	AB094402	partial	AB262975	AB262978
