# Supplementary figures and images for: Evolutionary history of the alpha2,8-sialyltransferase (ST8Sia) gene family: Tandem duplications in early deuterostomes explain most of the diversity found in the vertebrate ST8Sia genes
Source: BMC Evol Biol. 2008 Sep 23;8:258. doi: 10.1186/1471-2148-8-258 (PMC2564942; doi:10.1186/1471-2148-8-258)

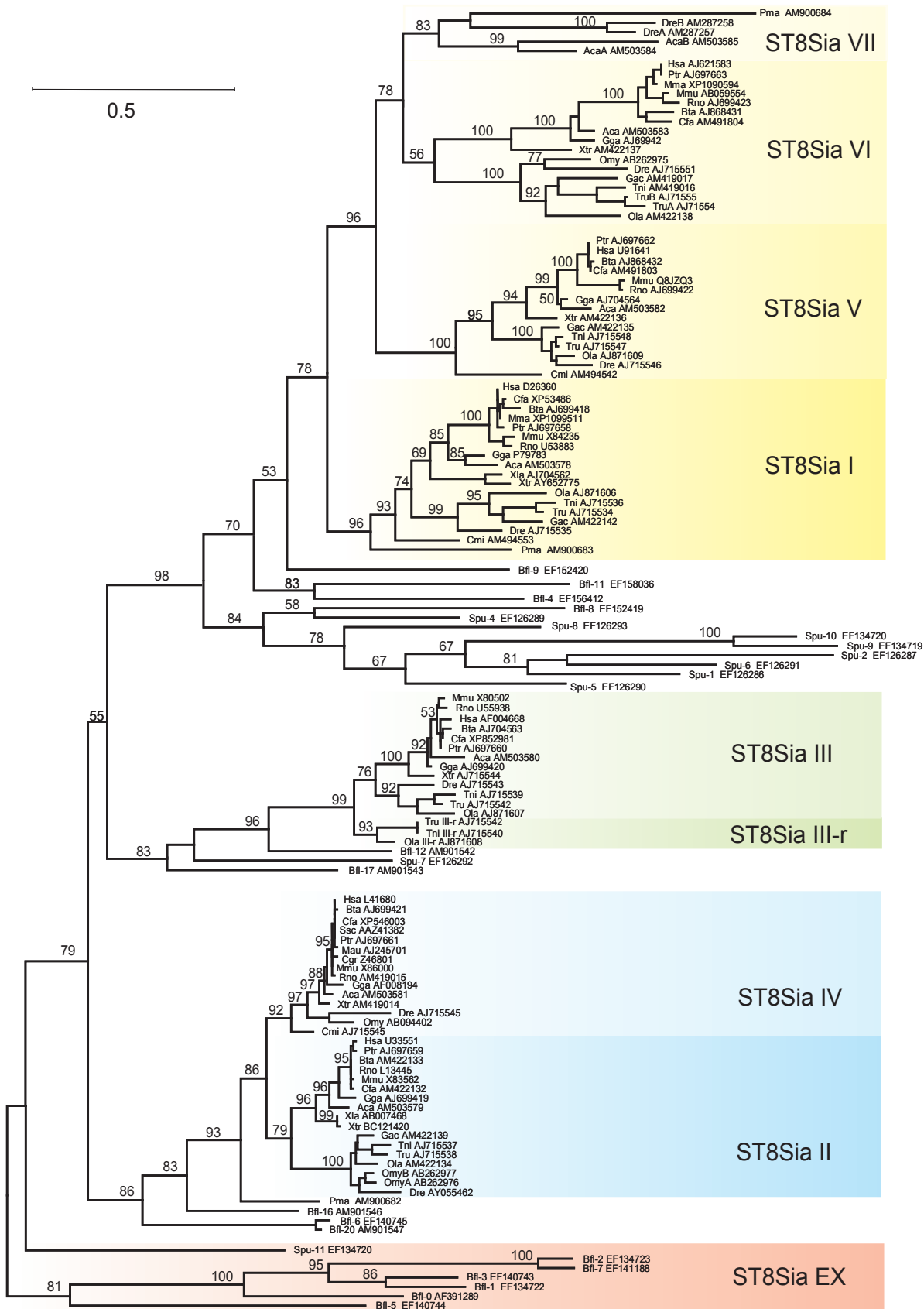

Supplement: Additional file 3 — Phylogenetic ML tree (PhyML software) with 129 sequences. One hundred and seventy three sites out of 521 positions (33%) within 10 informative blocks were identified with G-BLOCKS. Maximum Likelihood phylogenetic tree was constructed with Phyml, JTT model of amino acid substitution and 500 replicates. The mono-α2,8-sialyltransferases are yellow, the oligo-α2,8-sialyltransferases are green, the poly-α2,8-sialyltransferases are blue and the invertebrate ST8Sia EX sequences are pink. [file 1471-2148-8-258-S3.pdf]

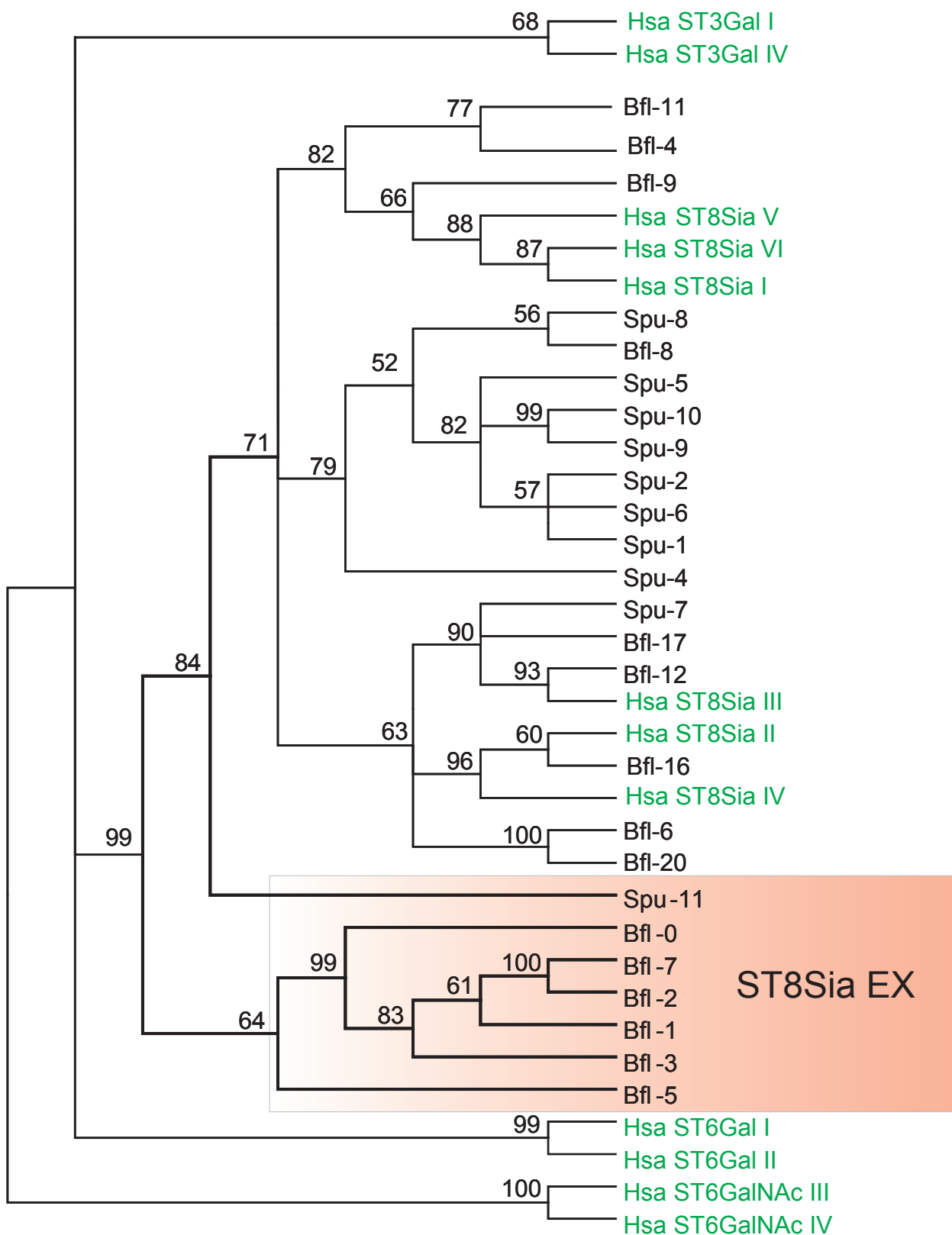

Supplement: Additional file 4 — Phylogenetic ML tree (PhyML software) with 12 human sialyltransferases and all the invertebrates ST8Sia sequences. One hundred and six sites out of 550 positions (19%) were selected with G-BLOCKS to find the most phylogenetically basal group within ST8Sia. User tree: topology obtained from Max likelihood with 500 replicates. The human ST6GalNAc III (AJ507291), ST6GalNAc IV (AJ271734), ST6Gal I (X17247), ST6Gal II (AB059555), ST3Gal I (L29555), ST3Gal IV (L23767) and the six human ST8Sia (ST8Sia I to ST8Sia VI) are indicated in green. All the invertebrate ST8Sia EX sequences are in a single red vanishing box. [file 1471-2148-8-258-S4.pdf]

**A**

ST8Sia II - ST8Sia IV

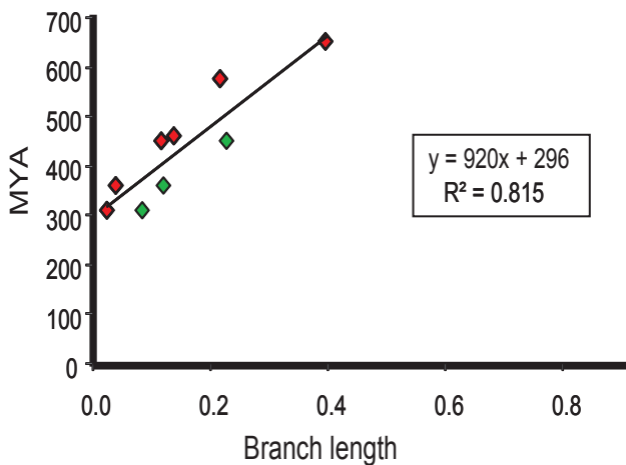**B**

ST8Sia III - ST8Sia III-r

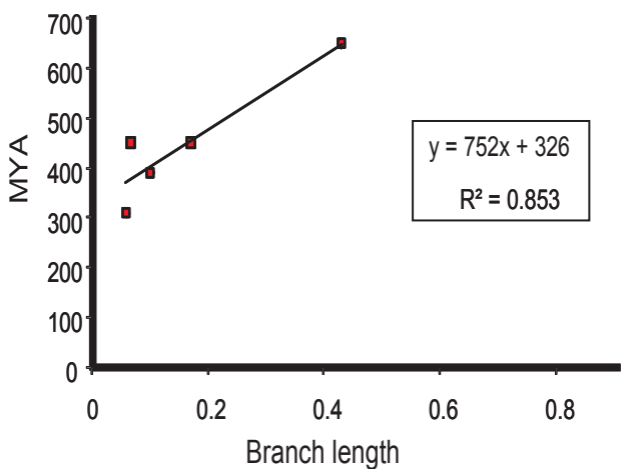**C**

ST8Sia I - ST8Sia V - ST8Sia VI - ST8Sia VII

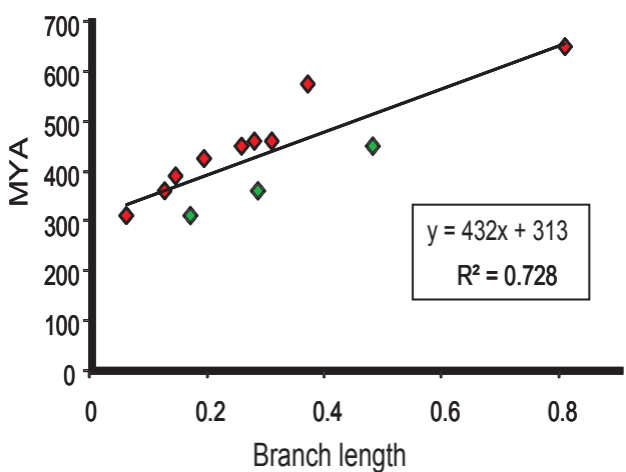

Supplement: Additional file 5 — Linear regression curves calculated for each group of sialyltransferases. A: poly-α2,8-sialyltransferase group (ST8Sia II and ST8Sia IV); B: oligo-α2,8-sialyltransferase group (ST8Sia III and ST8Sia III-r); C: mono-α2,8-sialyltransferases group (ST8Sia I, ST8Sia V, ST8Sia VI and ST8Sia VII). The equation of regression curves between the linearized branch lengths and datations in MYA are indicated in open boxes. Green diamonds refer to ST8Sia IV in graph A, and to ST8Sia VI in graph C. [file 1471-2148-8-258-S5.pdf]
